# A large outbreak of multiple* Salmonella* serovars linked to alfalfa sprouts in Norway, October to December 2024

**DOI:** 10.1007/s15010-025-02556-2

**Published:** 2025-05-21

**Authors:** Arthur Rakover, Liz E. Ødeskaug, Hilde Lund, Heidi Lange, Karina Kaupang, Taran O. Skjerdal, Laila Jensvoll, Bjarne Bergsjø, Polina Katsiouleri, Lamprini Veneti, Umaer Naseer, Lin T. Brandal, the Outbreak Investigation Team, Thale C Berg, Petter L Heradstveit, Silje B Lavoll, Tonje S Laird, Torbjørn Bruvik, Mark Woods, Elisabeth Madslien, Elisabeth Astrup, Monica Falk, Margrethe Hovda Røed, Turid Berglund, Gro Johannessen

**Affiliations:** 1 Norwegian Institute of Public Health, Oslo, Norway; 2 European Programme for Intervention Epidemiology Training (EPIET), European Centre for Disease Prevention and Control, (ECDC), Stockholm, Sweden; 3 The Norwegian Food Safety Authority, Oslo, Norway; 4 Norwegian Veterinary Institute, Oslo, Norway

**Keywords:** *Salmonella* outbreak, Sprouts, Whole genome sequencing, Case control study, Multiple serovars, Norway

## Abstract

**Purpose:**

This study investigates a nationwide *Salmonella* outbreak in Norway during October–December 2024 involving four different serovars—*S*. Newport, *S*. Typhimurium, *S*. Kisarawe, and *S*. Kinondoni. The investigation aimed to assess the outbreak's scope, identify the source, and implement control measures.

**Methods:**

Epidemiological analyses included trawling and targeted questionnaires, a matched case–control study, and grocery receipt analysis. Whole-genome sequencing (WGS) determined genetic links between *Salmonella* isolates from human cases, food, and environmental samples. Traceback investigations identified potential contamination sources.

**Results:**

A total of 230 cases (69% female, median age: 48 years) were identified, with 33% requiring hospitalization. Sprout consumption was reported by 69% of cases interviewed through trawling or targeted questionnaires. Grocery receipts were collected from some of the cases, and half of these had purchased sprouts. A matched case–control study found cases to be associated with consumption of sprouts (penalized adjusted odds ratio of 3.13). WGS established genetic links between clinical, food, and environmental isolates, identifying alfalfa sprouts as the outbreak source. Traceback investigations identified potential risk associated with seeds from an Italian supplier, previously associated with two *Salmonella* outbreaks in Norway in 2024 and multiple outbreaks across the European Union. The Italian supplier reported negative findings for *Salmonella* in their self-monitoring checks on seeds sent to Norway. Control measures included product withdrawal, seed batch quarantine, and public health advisories.

**Conclusion:**

This multi-serovar outbreak highlights the public health risks associated with consumption of raw sprouts and emphasizes the need for improved detection methods and stricter regulations to prevent future outbreaks.

**Supplementary Information:**

The online version contains supplementary material available at 10.1007/s15010-025-02556-2.

## Introduction

Salmonellosis remains a significant public health concern in the European Union and European Economic Area (EU/EEA), ranking as the second most frequently reported gastrointestinal infection among humans and a common cause of foodborne outbreaks [1, 2]. In Norway, between 2017 and 2019, the annual notification rate of salmonellosis cases ranged from 18.1 to 20.5 per 100,000 population [2]. While case numbers declined during the COVID-19 pandemic, they returned to pre-pandemic levels by 2024, with 1200 cases reported [3]. The predominant *Salmonella* serovars in Norway are *S.* Enteritidis and *S.* Typhimurium (including its monophasic variant) [4]. *Salmonella* is rarely detected in Norwegian livestock, locally produced feedstuffs, or food products. However, several nationwide outbreaks have been reported, mostly linked to imported products such as eggs, meat, vegetables and fruits [4–7]. In the summer of 2024, sprouts emerged as a public health concern in Norway after the Norwegian Institute of Public Health (NIPH) and the Norwegian Food Safety Authority (NFSA) observed two *Salmonella* outbreaks involving different serovars, both potentially linked to sprout consumption [8]. Since 2023, similar concerns regarding sprouts have been raised in several EU countries, and most recently in Sweden [9, 10]. While *Salmonella* outbreaks associated with fresh produce are well documented [11], outbreaks involving multiple serovars traced to a single food vehicle are rare and present notable epidemiological and microbiological challenges. Such events raise concerns about possible contamination pathways, hinder the establishment of clear case definitions, complicate source identification, and can delay the recognition and response to the outbreak.

Here, we present the findings of the outbreak investigation involving multiple *Salmonella* serovars linked to alfalfa sprouts in Norway between October and December 2024. The report aims to assess the outbreak’s scope, identify its source, and outline the control measures implemented to prevent further cases.

## Materials and methods

### Overview of the outbreak investigation and case definitions

In Norway, both clinicians and medical microbiological laboratories (MMLs) are obligated to report cases of *Salmonella* infection to the Norwegian Surveillance System for Communicable Diseases (MSIS). In addition, MMLs have to submit *Salmonella* isolates to the National Reference Laboratory (NRL) for Enteropathogenic Bacteria at the NIPH for confirmation and molecular epidemiological surveillance by whole genome sequencing (WGS) [4].

**Fig. 1:**
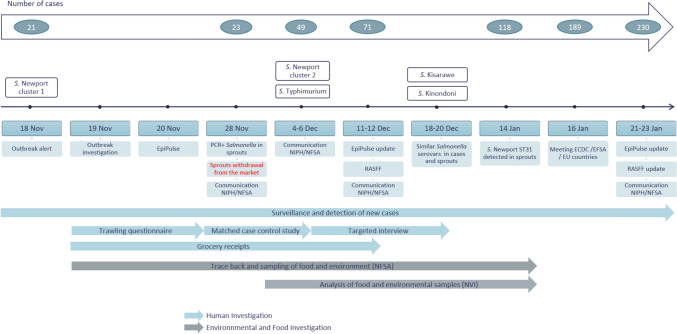
Timeline of the national multi-serovar *Salmonella* outbreak linked to alfalfa sprouts in Norway, October–December 2024

On 18 November 2024, the NRL for Enteropathogenic Bacteria reported a cluster of 21 cases of *S.* Newport sequence type (ST) 31 and cluster type (CT) 25548 to NIPH. In response, NIPH initiated an outbreak investigation in collaboration with NFSA, the Norwegian Veterinary Institute (NVI), and the relevant municipal medical officers. Following the notification, all cases reported with *S*. Newport ST31 with CT25548 were included in the outbreak. To investigate potential sources of infection, the first cases of the initial *S*. Newport cluster (cluster 1) were interviewed using a hypothesis-generating trawling questionnaire, while some of the subsequent cases in the same cluster were enrolled in a case–control study. In the following weeks, additional cases caused by a new *S*. Newport cluster (cluster 2) and by other *Salmonella* serovars—including *S. Typhimurium*, *S. Kisarawe*, and *S. Kinondoni*—were reported. Epidemiological and microbiological evidence indicated that these cases were linked to the same outbreak and likely shared a common source of exposure (Fig. 1). As a result, these serovars were incorporated into the case definition.

An outbreak case was defined as a person living in Norway with symptom onset after 1 October 2024 and a laboratory-confirmed infection with one of the following *Salmonella*:

- *S.* Newport ST31 and CT25548 (cluster 1) or CT26172 (cluster 2) with ≤ 5 allelic differences (AD) within each cluster.
- *S.* Typhimurium ST36 clustering with one of 14 outbreak strains from Sweden with ≤ 10 AD.
- *S.* Kisarawe ST5805 clustering with one of the outbreak strains detected in alfalfa sprouts with ≤ 20 AD.
- *S*. Kinondoni ST5447 clustering with one of the outbreak strains detected in alfalfa sprouts with ≤ 10 AD.

### Epidemiological investigation

#### Trawling questionnaire

To generate hypotheses about the source of infection, the NFSA conducted interviews with initial *S*. Newport cluster 1 cases using a standardized trawling questionnaire for *Salmonella* investigations. The questionnaire collected information on symptoms, date of onset, travel history, contact with animals and food consumption during the week prior to symptom onset [12].

#### Case-control study

We conducted a case–control study to test preliminary hypotheses generated from the trawling questionnaire regarding the potential source of the outbreak among *S*. Newport cluster 1 cases. In order to independently assess the hypotheses generated from the trawling questionnaire we did not include cases already interviewed in the case–control study. Controls were randomly selected from the Norwegian population register, with three controls matched per case based on age, sex, and municipality of residence. Both cases and controls were interviewed by NIPH regarding their consumption of products identified as potential outbreak sources through the trawling questionnaires. Data were collected on the consumption of sprouts, chicken, arugula, grapes, and leeks, focusing on the week preceding symptom onset for the cases and in a typical week in the second half of October for the controls. Controls were excluded if they had traveled abroad for more than a week during the second half of October or had experienced acute diarrhea (defined as three or more loose stools per day) after mid-October 2024. We performed univariable analysis using conditional logistic regression and calculated odds ratios (ORs) with 95% confidence intervals (CIs) for food exposures. We performed multivariable analysis using Lasso regression to estimate penalized adjusted odds ratios (aORs). All data analyses were performed using R version 4.4.0.

#### Targeted interviews and MSIS reporting

Building on the findings from the trawling questionnaires and the case–control study, targeted interviews were conducted to gather detailed information from new cases—including serovars *S.* Newport cluster 2, *S.* Typhimurium, *S.* Kisarawe, and *S.* Kinondoni— regarding the consumption of food items identified as potential outbreak source, with a particular focus on sprout consumption. Additionally, the MSIS notifications submitted by physicians and MMLs include personal details, clinical information, laboratory results, relevant travel history, and, when available, information on food consumption. For this investigation, we reviewed data on suspected sources of infection as reported by clinicians, based on the optional free-text fields included in the MSIS notification reports.

#### Grocery receipts analysis

NIPH and NFSA obtained consent from interviewed cases to collect grocery receipts for the two weeks preceding symptom onset. We gathered receipts through direct submission from cases, loyalty card numbers, or online banking records. The collected data were analyzed to identify food items consistent with the primary hypothesis.

### Microbiological investigation

#### Human cases

We performed WGS on all *Salmonella* isolates received at the NRL using paired-end sequencing on the NextSeq platform (Illumina, Inc., San Diego, US), targeting a coverage of > 50×. All sequences have been submitted to the European Nucleotide Archive (ENA) and are available through BioProject PRJEB65328.

Sequences were assembled using SPAdes v3.13.0, and species and serotypes were determined using an in-house pipeline [13]. Raw sequence reads were imported into Ridom SeqSphere+ v10.0.1 and sequence type and cluster type were assigned as previously described [14]. *S*. *enterica* serovars were identified using SISTR Geno-Serotyping. The allelic profiles of the isolates were visualized as a minimum spanning tree (MST) using the ‘pairwise ignoring missing values’ parameter. Sequences from *Salmonella* detected in environmental and food samples, provided by NVI, were compared with human isolates in the national genome database, which includes all human *Salmonella* sequences from 2018 onwards. An outbreak cluster was defined as two or more isolates with ≤ 5, ≤ 10, or ≤ 20 AD, dependent on serovar, detection of isolates in sprouts, and comparison with Swedish *S*. Typhimurium outbreak strains.

#### Environmental and food samples

NFSA collected food samples of suspected alfalfa sprouts, all traced back to a single Norwegian sprout producer (Producer A). Samples included sealed packages, opened packages from patients' homes, sprouts from a buffet tray, and environmental samples from Producer A's facility. All *Salmonella* isolates from non-human sources were sent to the National Reference Laboratory for *Salmonella* (NRL-*Salmonella*) at NVI. Microbiological analyses were conducted using VIDAS® UP Salmonella SPT (bioMerieux) and ISO 6579-1:2017 (Supplementary Appendix 1). Isolates were serotyped and WGS was performed. Sequences from *Salmonella* found in sprouts or environmental samples were shared with NIPH for comparison with human sequences and submitted to ENA (PRJEB85723).

### Trace-back investigation

Based on epidemiological findings from trawling questionnaire and grocery receipt analysis, the NFSA conducted a trace-back investigation of the implicated products. Given the strong suspicion surrounding sprouts, the NFSA met with Producer A on 26 November to inform them of the concerns and implement control measures at the production facility.

### International investigation

On 19 November, NIPH posted the event in EpiPulse (2024-FWD-00116), shared fasta and raw reads of *S*. Newport cluster 1 to inquire whether other countries had observed cases with the outbreak strain. On 12 December, NIPH updated the event with sequences of *S*. Newport ST31 cluster 2 and reported cases of *S.* Typhimurium ST36 linked to a Swedish outbreak (2024-FWD-00108). A subsequent update on 25 January included sequences for *S*. Kisarawe and *S*. Kinondoni. Throughout the investigation, Norwegian authorities maintained close collaboration with their Swedish counterparts.

## Results

### Epidemiological investigation

#### Descriptive epidemiology

**Fig. 2:**
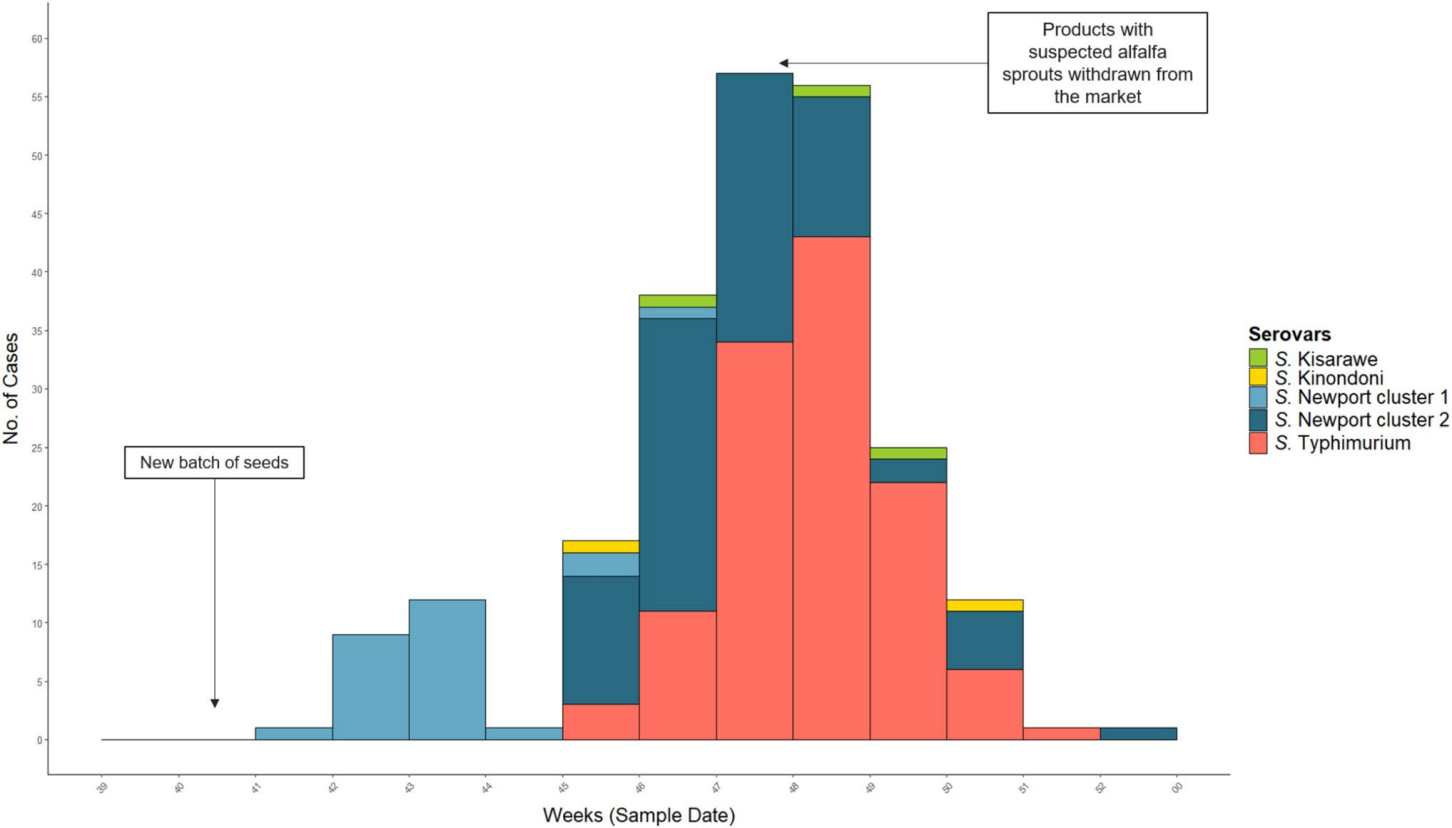
Epidemic curve of the national multi-serovar *Salmonella* outbreak linked to alfalfa sprouts by sampling week, Norway, October–December 2024^a^. ^a^Among 230 cases, *S.* Typhimurium was most common (120 cases, 52.2%), followed by *S.* Newport cluster 2 (79 cases, 34.3%) and cluster 1 (26 cases, 11.3%). *S.* Kisarawe and *S.* Kinondoni were rare, with 3 (1.3%) and 2 (0.9%) cases, respectively

As of 21 January 2025, a total of 230 *Salmonellosis* cases were linked to the national outbreak including four serovars. Symptom onset dates were available for 94 cases (53%), ranging from 11 October to 18 December 2024, with the majority occurring before 3 December. Sampling dates spanned from 16 October to 30 December 2024 (Fig. 2). The median age of cases was 48 years (range: 10 months–90 years), and 159 (69%) were female (Supplementary Table S1 and Fig. S1). Cases were reported across all counties of Norway (Supplementary Fig. S2), and 76 individuals (33%) required hospitalization.

#### Trawling questionnaire

Ten *S*. Newport cluster 1 cases were interviewed with a trawling questionnaire (Table 1). Food items frequently reported by cases were sprouts (7, two unsure for exposure), grapes (7), chicken breast (6, one unsure), arugula (5, two unsure), and leeks (5, one unsure).

**Table 1:** Summary of epidemiological investigation findings in the national multi-serovar *Salmonella* outbreak linked to alfalfa sprouts, Norway, October–December 2024

Serovars	Cases	Sprout consumption among interviewed cases			Sprout purchase among grocery receipts collected^a^	Sprout consumption reported in MSIS^b^
		Trawling questionnaire	Case–control study	Targeted questionnaire		
*S*. Newport cluster 1	26	7/10 (70%)^c^	8/12 (67%)	–	6/13 (46%)	–
*S*. Newport cluster 2	79	–	–	5/8 (63%)	2/3 (67%)	6
*S*. Typhimurium	120	–	–	3/5 (60%)	1/1 (100%)	35
*S*. Kisarawe	3	–	–	2/2 (100%)	–	1
*S*. Kinondoni	2	–	–	1/1 (100%)	–	–
Total	230	26/38 (68%)			9 /17 (53%)	42

^a^Grocery receipts were collected from cases interviewed with trawling or targeted questionnaire

^b^Clinicians reported sprouts in MSIS as suspected source of infection (optional free text in notification report)

^c^Additionally, 20% (2/10) replied that they were not sure about their consumption sprouts

#### Case–control study

The case–control study included 47 participants, comprising 12 *S*. Newport cluster 1 cases (26%) and 35 controls (Table 2). The median age of participants was 48 years (range 18–63 years), and 31 (66%) were women. In univariable analysis, no significant differences were found between cases and controls regarding the consumption of chicken breast, grapes, leeks, or arugula. Univariate conditional logistic regression did not converge for sprouts due to small number of controls reporting sprout consumption. Sprout consumption was reported from 67% of cases (8/12) compared to only 3% of controls (1/35). In the multivariable model using Lasso regression, cases were three times more likely to have consumed sprouts than controls (penalized adjusted OR:3.13).

**Table 2:** Results from a matched case control study regarding food exposures in the national multi-serovar *Salmonella* outbreak in Norway, October–December 2024 (cases = 12, controls = 35)

Food items	Cases (N = 12)		Control (N = 35)		Crude Odds Ratio^a^	Penalized adjusted odds ratio^b^
	No /total exposed	% exposed	No /total exposed	% exposed		
Sprouts	8/12	67%	1/35	3%	N/A^c^	3.13
Grapes	4/12	33%	21/35	60%	0.36 (0.09–1.45)	1
Leeks	3/12	25%	16/35	46%	0.40 (0.09–1.74)	1
Aragula	8/12	67%	18/35	51%	1.76 (0.47–6.51)	1
Chicken breast	6/12	50%	20/35	57%	0.78 (0.22–2.78)	1

^a^Univariable conditional logistic regression

^b^Obtained using LASSO regression, which performs variable selection but does not give confidence intervals. Adjusted ORs that are not equal to 1 are statistically significant

^c^Univariate conditional logistic regression did not converge

#### Targeted interviews and MSIS reporting

A total of 16 cases were interviewed using targeted questionnaires. Among them, sprout consumption was reported by 63% (5/8) of *S*. Newport cluster 2 cases, 60% (3/5) of *S*. Typhimurium cases, all cases of *S*. Kisarawe (3/3), and 50% of *S*. Kinondoni cases (1/2). Additionally, sprout consumption was reported for 42 cases through MSIS (Table 1).

#### Grocery receipts analysis

We received receipts from 17 cases. Analysis confirmed that six *S*. Newport cluster 1 cases, two *S*. Newport cluster 2 cases, and one *S*. Typhimurium case had purchased alfalfa sprouts from Producer A (Table 1).

### Microbiological investigation

#### Human cases

**Fig. 3:**
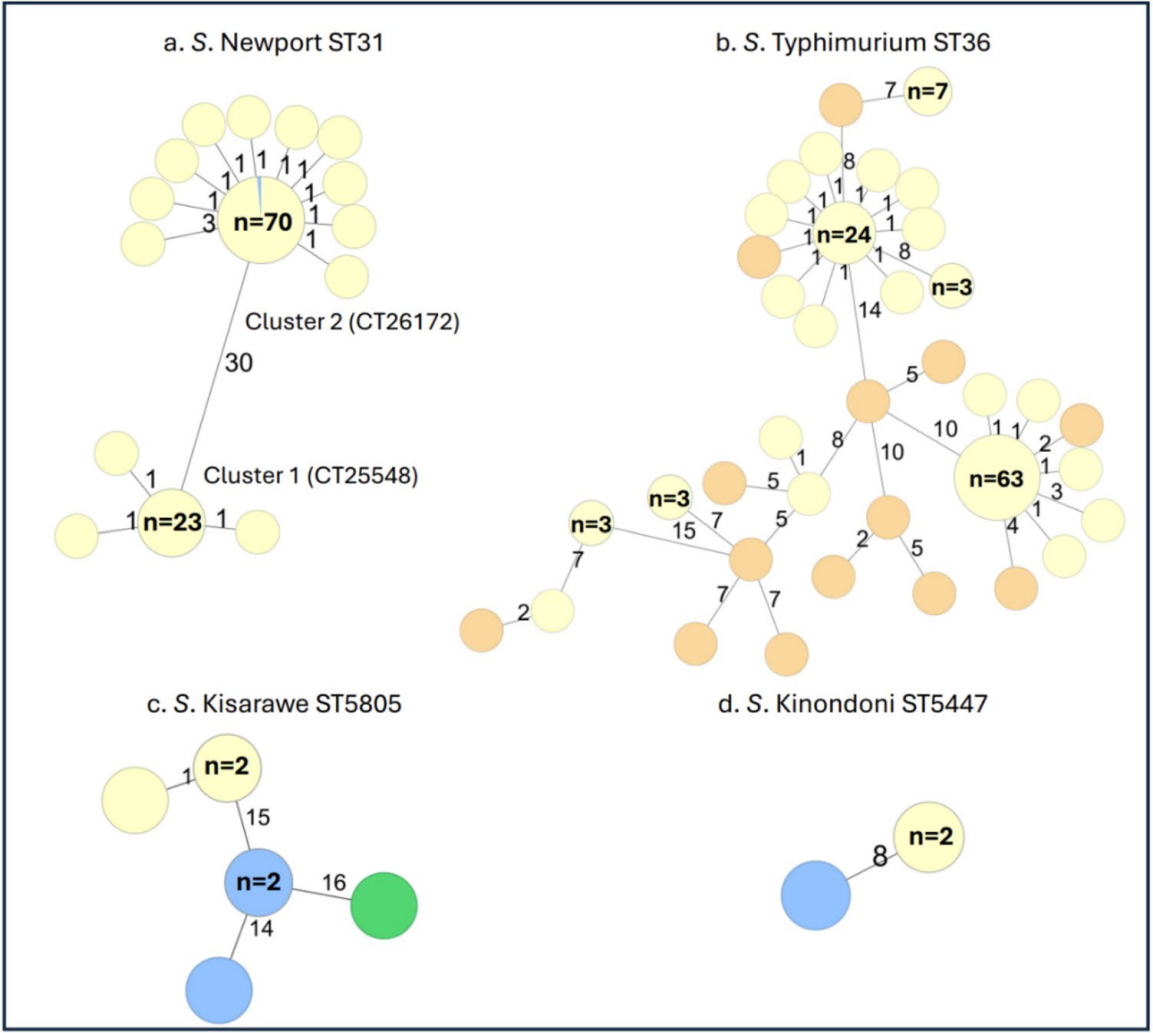
Minimum spanning tree of *Salmonella* serovars associated with the alfalfa sprouts outbreak in Norway, October–December 2024, based on core genome multilocus sequence typing (cgMLST) analysis^a^. ^a^Numbers on branches in the minimum spanning tree indicate the number of allelic differences between isolates. The number of isolates is indicated in each circle. Isolates from Norwegian human cases (n = 230: 105 *S.* Newport, 120 *S.* Typhimurium, 3 *S.* Kisarawe, and 2 *S.* Kinondoni) are in yellow. Isolates from the Swedish outbreak (n = 14, 2024-FWD-00108) are in orange. Isolates from sprouts (n = 5): 1 from a sealed package (*S.* Kisarawe), 2 *Salmonella* isolates from a single buffet tray sample (*S.* Kisarawe and *S.* Kinondoni), and 2 from open packages linked to ill persons (*S.* Newport cluster 2 and *S.* Kisarawe), all from Producer A, are in blue. The environmental isolate from Producer A (n = 1) is in green

We identified four different *Salmonella* serovars potentially linked to alfalfa sprouts, involving in total 230 cases: 105 cases of *S.* Newport ST31, 120 cases of *S*. Typhimurium ST36, 3 cases of *S*. Kisarawe ST5805, and 2 cases of *S*. Kinondoni ST5447. For *S.* Newport ST31, two distinct clusters were identified: CT25548 (cluster 1) and CT26172 (cluster 2), with 30 AD between them and fewer than 5 AD within each cluster (Fig. 3a). *S*. Typhimurium ST36 isolates from Norway clustered with fewer than 10 AD to the 14 Swedish outbreak strains reported in EpiPulse (Fig. 3b). *S*. Kisarawe and *S*. Kinondoni are rare serovars not previously observed in the national genome database. During the outbreak investigation, cases with *S*. Kisarawe and *S*. Kinondoni were found to cluster with *Salmonella* from alfalfa sprouts with fewer than 20 AD and 10 AD, respectively (Fig. 3c and d).

#### Environmental and food samples

A total of eight *Salmonella* isolates were recovered from seven samples collected by the NFSA during the outbreak investigation, including sprouts and environmental samples from Producer A (Table 3). Additionally,* S*. Kotte was isolated from sprouts from Producer A by a distributor's own control. Sealed packages of sprouts tested positive for three *Salmonella* serovars: *S*. Kotte,* S*. Newport ST166 (2), and *S*. Kisarawe. Opened packages were positive for: *S*. Kinondoni, *S*. Kisarawe (2), and *S*. Newport ST31. One environmental sample was positive for *S*. Kisarawe. No human isolates clustered with *S*. Kotte or *S*. Newport ST166 detected in sprouts. However, *S*. Newport ST31 from sprouts was identical to the human *S*. Newport ST31 cluster 2 (0 AD). Within the *S*. Kisarawe cluster, 14–16 AD were identified among isolates from sprouts, the environment, and human cases, while only 1 AD was observed between the human *S*. Kisarawe cases (Fig. 3c). Similarly, for *S*. Kinondoni, 8 AD were found between human cases and sprouts, whereas isolates from the human cases were identical (Fig. 3d).

**Table 3:** Detection of *Salmonella* in sprouts and environmental samples during the national multi-serovar *Salmonella* outbreak linked to alfalfa sprouts in Norway, October–December 2024

Food/Environmental samples	Sampling date	Packing date	Results (serovar and sequence type)	Clustering with human cases
Sealed packages	27/11/2024	26/11/2024	*S*. Kotte (ST11989)	No
	28/11/2024	22/11/2024	*S*. Newport (ST166)^a^	No^e^
	28/11/2024	19/11/2024	*S*. Kisarawe (ST5805)	Yes
Opened packages	28/11/2024	Not available	*S*. Kinondoni (ST5447)^b^	Yes
	28/11/2024	Not available	*S*. Kisarawe (ST5805)^c^	Yes
	10/12/2024	19/11/2024	*S*. Kisarawe (ST5805)^c^	Yes
	13/12/2024	01/11/2024	*S*. Newport (ST31)^c^	Yes
Producer A facility	28/11/2024	Not available	*S*. Kisarawe (ST5805)^d^	Yes

^a^Two isolates from the same lot, both sequenced (*S*. Newport ST166 with 0 AD between the isolates)

^b^Alfalfa sprouts from a buffet tray. One sample with two *Salmonella* serovars

^c^Opened packages from two different private households

^d^Swab of "run-off" water from a Rota-Teck rotary drum after 21 h of operation

^e^*S*. Newport ST166 found in sprouts differed genetically from the* S*. Newport ST31 isolates obtained from patients by more than 2,500 alleles, indicating no epidemiological link

### Trace-back investigation

Producer A received two seed batches from an Italian supplier (Supplier A): one used from February to September 2024 and another from October to 28 November 2024. Throughout the use of these batches, Producer A conducted routine sampling and microbiological testing in compliance with Regulation 2073/2005, with no *Salmonella* detected. However, on 28 November 2024, a distributor of sprouts from Producer A reported a positive PCR result for *Salmonella* following testing based on suspicion. An inspection by NFSA confirmed that Producer A was in full compliance with hygiene Regulations 852/2004. A Norwegian RASFF alert (2024.9155), was issued on 10 December 2024, notifying authorities of the suspected link to alfalfa seeds from Italy. The Supplier A responded that all batches of seed had been tested without any positive *Salmonella* findings.

### International investigation

Of the nine countries responding to the EpiPulse notification, only one reported cases clustering with the *Salmonella* outbreak strains identified in Norway (2024-FWD-00116, later merged into 2025-FWD-00006). Sweden identified four cases of *S*. Kinondoni clustering with Norwegian cases; however, their investigation found no epidemiological evidence of sprout consumption. Sweden also reported a concurrent outbreak of* S*. Typhimurium involving 100 cases linked to alfalfa sprouts, with the Norwegian *S*. Typhimurium outbreak strains clustering within the Swedish outbreak (2024-FWD-00108). Traceback investigations carried out in both countries identified Supplier A as the common source.

### Outbreak control measures

On 28 November 2024, products containing the suspected alfalfa sprouts were withdrawn from the market, and the remaining alfalfa seeds in Norway were placed under quarantine. To eliminate any potential contamination risks, decontamination of equipment and the production facility at Producer A was conducted. Both Producer A and NFSA implemented intensified sampling measures. To maintain public awareness, NIPH and NFSA issued multiple updates on the outbreak, product withdrawal, and consumer advice (Fig. 1) [15, 16]. This information was also disseminated through national media to ensure broad public reach. Meanwhile, NIPH continued routine surveillance of *Salmonella* cases, to monitor the outbreak and detect new cases. At the request of Sweden and Norway, the European Centre for Disease Prevention and Control (ECDC), in collaboration with the European Food Safety Authority (EFSA), organized a meeting with member states and the European Commission, to coordinate the outbreak response and develop a Joint Rapid Outbreak Assessment (ROA) [17].

## Discussion

We describe the largest *Salmonella* outbreak in Norway since the 1980s, with 230 cases and multiple serovars detected across the country between October and December 2024. Comprehensive epidemiological, microbiological, and traceback investigations identified alfalfa sprouts as the outbreak source. These sprouts were produced by a specific Norwegian facility (Producer A) using seeds supplied by an Italian seed supplier (Supplier A).

Earlier in 2024, two additional *Salmonella* outbreaks in Norway were linked to sprout consumption. The first batch from Supplier A, used between February and September 2024, was suspected to be associated with these outbreaks—one from May to August with *S*. Typhimurium ST36 and another from August to October with *S*. Hvittingfoss. The second batch, used from October until 28 November, was linked to the current *Salmonella* outbreak. Since 2023, multiple RASFF alerts and EpiPulse notifications have associated sprouts consumption with *Salmonella* outbreaks across the EU/EEA, affecting countries such as Sweden, Finland, Spain, Germany, Italy, and Norway (Supplementary Table S2) [17, 18]. By sharing data from independent national outbreak investigations, the joint ECDC-EFSA ROA revealed that all outbreaks were linked to a common alfalfa seed supplier in Italy (Supplier A), which sourced seeds from three seed growers in the same geographical region of Italy [17].

The detection of multiple *Salmonella* serovars—such as *S*. Newport, *S*. Typhimurium, *S*. Kisarawe, and *S*. Kinondoni—within a single outbreak is highly unusual and highlights the complexity of this event. The diversity of serovars suggests that the contamination of the alfalfa sprouts was not due to a single contamination event but likely resulted from multiple contamination points or sources along the production and supply chain. A similar scenario was observed in a previous multi-country outbreak linked to a sesame-based product imported from Syria, where multiple *Salmonella* serovars were also identified [6]. Multi-serovar outbreaks pose significant challenges for public health surveillance and outbreak detection. They can be often missed or underestimated when surveillance systems focus on a single serovar or genetic cluster and fail to integrate well epidemiological and microbiological data. This highlights the need of using advanced molecular tools, such as WGS, which can identify and link genetically diverse isolates to a common source of exposure. It also emphasizes the importance of interpreting WGS data in conjunction with epidemiological findings to accurately identify the source of the outbreak. Additionally, these outbreaks underscore the importance of having flexible outbreak investigation criteria and greater awareness of complex contamination scenarios, particularly in high-risk food products such as sprouted seeds.

Sprouts are a well-known source of foodborne illness, often implicated in *Salmonella* outbreaks [19–22]. In 2007, Norway, Sweden, and Finland experienced an outbreak with *S*. Weltevreden associated with consumption of alfalfa sprouts. Traceback investigations suggested that contaminated seeds, likely originating from Italy, were the source of the infection [7]. Since 1988, sprout consumption has been associated with over 60 outbreaks worldwide, many of which were specifically traced to alfalfa sprouts [19]. Sprouts pose a high-risk due to the warm and humid conditions required for their growth, which also favor bacterial proliferation [23]. Moreover, because alfalfa sprouts are typically consumed raw, pathogens present are not eliminated by cooking, increasing the potential for human infection [19–22].

Seed contamination can occur at various stages, ranging from field cultivation to post-harvest handling, storage, and distribution. Environmental factors—such as the use of contaminated irrigation water, exposure to tainted soil, animal intrusion, and poor hygiene practices during harvesting and processing—can all contribute to the introduction of pathogens [24]. Once seeds are contaminated, bacteria can persist and even multiply throughout the sprouting process, making early-stage contamination prevention critical to food safety [25]. The EU has a comprehensive regulatory framework for sprouts, requiring all food business operators (FBOs) to adhere to general hygiene standards (Regulation 852/2004) and microbiological criteria (Regulation 2073/2005), implement good agricultural and manufacturing practices, and routinely test for pathogens like *Salmonella* [26, 27]. No deviations were found at Producer A. All pre-outbreak samples taken by the producer were negative for *Salmonella*, but several samples taken during the outbreak by NFSA were positive for different *Salmonella* serovars. Posted RASFF alerts from various countries also reported *Salmonella* detected in sprouts through official testing. Contrary, the Supplier A only reported negative findings for *Salmonella* in their self-monitoring checks on seeds sent to Norway and Sweden. The joint ECDC-EFSA ROA also highlighted the need for further investigation into the role of environmental factors in seed contamination at the grower level, as seeds to Supplier A were received from three seed growers in the same geographical area of Italy, as well as potential cross-contamination along the seed supply chain [17].

Our investigation suggests that reinforcing official controls by the local competent authority overseeing the Supplier A involved in this outbreak is crucial. Inconsistencies in detecting *Salmonella* in environmental and seed samples posed significant challenges. The challenge of obtaining representative samples from an entire seed lot—sometimes weighing several tons—makes it difficult to detect contamination, especially as it may be unevenly distributed. The detection of *Salmonella* is highly dependent on sampling strategies, and seeds and sprouts are complex matrices that can challenge detection methods. Therefore, detection methods should be validated specifically for this matrix. In the current investigation, two analytical methods were used to increase the likelihood of detection. Two of the isolates were detected with one method and the remaining six with the other. When Supplier A reported no detection of *Salmonella*, it may reflect limitations of the testing regimen used rather than an actual absence of contamination. Improved sampling protocols and appropriate methods of testing are essential for detection of *Salmonella* contamination. Revisiting regulatory framework for sprout production and methods used for detecting microbiological contamination could significantly improve food safety measures and reduce the risk of future outbreaks.

This outbreak also underscores the public health risks associated with internationally traded seeds and the critical need for coordinated cross-border response. The detection of multiple *Salmonella* strains in several EU/EEA countries, all linked to the same seed supplier, highlights the importance of timely communication through platforms such as EpiPulse and RASFF, and systematic sharing of WGS, epidemiological, and traceability data across public health and food safety sectors. These collaborative efforts, culminating in the joint ECDC-EFSA ROA were instrumental in identifying the common source, reinforcing the value of international cooperation.

Our investigation employed a comprehensive, multidisciplinary approach that integrated epidemiological, traceback, and microbiological methods, with WGS playing a key role in linking human cases to the contaminated sprout samples in Norway and to the Swedish *S*. Typhimurium outbreak strains. Several *Salmonella* serovars were detected in sprouts, with strains within the same serovar varying in genetic distance—from 0 AD for *S*. Kisarawe to over 2500 AD for *S*. Newport ST166 and ST31, indicating extensive contamination of the sprouts. While the typical threshold to define *Salmonella* isolates in an outbreak is 2–5 AD [28], we expanded this threshold to 10–20 AD within each serovar in this outbreak to capture all potential sources of contamination.

Despite these strengths, some limitations impacted our investigation. As always in case–control studies and interviews, recall bias could be present, as participants sometimes would struggle to remember whether they had consumed sprouts, particularly when they were included in meals like sandwiches or salads. This may have resulted in misclassification of exposure status, potentially biasing the estimated odds ratios. Another important limitation is the potential bias in case identification inherent in the passive nature of the laboratory surveillance system. The investigation likely captured predominantly severe cases, with milder or moderate cases potentially going undetected. Both these limitations have likely resulted in under-reporting of cases, affecting our ability to fully characterizing the extent of the outbreak.

## Conclusion

Alfalfa sprouts present a significant public health risk, particularly in Nordic countries where they are often consumed raw. Raising public awareness is crucial. NIPH and NFSA should continue to provide timely guidance on the risks associated with raw sprout consumption, particularly advising vulnerable groups, such as children, immunocompromised individuals, and pregnant women, to avoid raw sprouts. At the national level, NIPH should enhance real-time surveillance of foodborne pathogens, while NFSA should implement more frequent official controls, including increased sampling of food business operators involved in sprout production. At the international level, recognizing alfalfa sprouts as a high-risk product could lead to ECDC and EFSA conducting comprehensive risk assessments and revising regulations on sprout testing and safety, ultimately improving protection of public health.

## Supplementary Information

Below is the link to the electronic supplementary material.

Supplementary file1 (DOCX 89 KB)

## Data Availability

No datasets were generated or analysed during the current study.

## Declarations

### Use of artificial intelligence tools

None declared.

## References

[1] European Centre for Disease Prevention and Control. Salmonellosis. In: ECDC. Annual Epidemiological Report for 2022. Stockholm: ECDC; 2024.

[2] European Food Safety Authority (EFSA), European Centre for Disease Prevention and Control (ECDC). The European Union One Health 2023 Zoonoses report. EFSA J. 2024;22. 10.2903/j.efsa.2024.9106 PMC11629028 39659847

[3] FHI MSIS statistikkbank [Internet]. https://allvis.fhi.no/msis.

[4] Salmonellose [Internet]. 2024. https://www.fhi.no/sm/smittevernhandboka/sykdommer-a-a/salmonellose/.

[5] Johansen TB, Brandal LT, MacDonald E, Naseer U, Stefanoff P, Røed MH, et al. Exotic dried fruits caused Salmonella Agbeni outbreak with severe clinical presentation, Norway, December 2018 to March 2019. Eurosurveillance. 2021;26. 10.2807/1560-7917.ES.2021.26.14.2000221 PMC8034060 33834962

[6] European Food Safety Authority (EFSA). Multi‐country outbreak of multiple Salmonella enterica serotypes linked to imported sesame‐based products. EFSA Support Publ. 2021;18.

[7] Emberland KE, Ethelberg S, Kuusi M, Vold L, Jensvoll L, Lindstedt BA, et al. Outbreak of Salmonella Weltevreden infections in Norway, Denmark and Finland associated with alfalfa sprouts, July-October 2007. Wkly Releases 1997–2007. 2007;12. 10.2807/esw.12.48.03321-en 18053569

[8] Alfalfaspirer mistenkt som smittekilde i nasjonalt utbrudd av Salmonella [Internet]. 2024. https://www.fhi.no/nyheter/2024/alfalfaspirer-mistenkt-som-smittekilde-i-nasjonalt-utbrudd-av-salmonella/.

[9] Norway reports largest outbreak in decades with 230 sick; sprouts blamed [Internet]. https://www.foodsafetynews.com/2025/01/norway-reports-largest-outbreak-in-decades-with-230-sick-sprouts-blamed/.

[10] Salmonella Typhimurium ST 36 (Sverige, augusti–december 2024) [Internet]. 2024. https://www.folkhalsomyndigheten.se/smittskydd-beredskap/utbrott/utbrottsarkiv/salmonella-typhimurium-st-36-sverige-augusti-2024/.

[11] Hanning IB, Nutt JD, Ricke SC. Salmonellosis outbreaks in the United States due to fresh produce: sources and potential intervention measures. Foodborne Pathog Dis. 2009;6:635–48. 19580447 10.1089/fpd.2008.0232

[12] Spørreskjema, retningslinjer og andre hjelpemidler [Internet]. https://www.fhi.no/ut/utbruddshandboka/hjelpemidler/sporreskjema-og-retningslinjer/.

[13] Garcia N. Github: garcia-nacho/TOP [Internet]. 2025. https://github.com/garcia-nacho/TOP.

[14] Siira L, Naseer U, Alfsnes K, Hermansen NO, Lange H, Brandal LT. Whole genome sequencing of Salmonella Chester reveals geographically distinct clusters, Norway, 2000 to 2016. Eurosurveillance. 2019;24. 10.2807/1560-7917.ES.2019.24.4.1800186 PMC6352000 30696528

[15] Utbrudd av salmonellose (salmonellainfeksjon) [Internet]. 2024. https://www.fhi.no/ut/utbrudd/oversikt-over-storre-utbrudd/utbrudd-av-salmonellose/.

[16] Alfalfaspirer mistenkt smittekilde i nasjonalt utbrudd av salmonella | Mattilsynet [Internet]. https://kommunikasjon.ntb.no/pressemelding/18341111/alfalfaspirer-mistenkt-smittekilde-i-nasjonalt-utbrudd-av-salmonella?publisherId=10773547&lang=no.

[17] European Food Safety Authority. Prolonged cross‐border multi‐serovar Salmonella outbreak linked to consumption of sprouted seeds.

[18] RASFF Window—Search [Internet]. https://webgate.ec.europa.eu/rasff-window/screen/search.

[19] Miyahira RF, Antunes AEC. Bacteriological safety of sprouts: a brief review. Int J Food Microbiol. 2021;352: 109266. 34111728 10.1016/j.ijfoodmicro.2021.109266

[20] Harfield S, Beazley R, Denehy E, Centofanti A, Dowsett P, Housen T, et al. An outbreak and case-control study of Salmonella Havana linked to alfalfa sprouts in South Australia, 2018. Commun Dis Intell. 2019;43. 10.33321/cdi.2019.43.45 31610770

[21] Mahon BE, Pöunkä A, Hall WN, Komatsu K, Dietrich SE, Siitonen A, et al. An international outbreak of *Salmonella* infections caused by alfalfa sprouts grown from contaminated seeds. J Infect Dis. 1997;175:876–82. 9086144 10.1086/513985

[22] Werner S, Boman K, Einemo I, M Erntell, R Helisola, B de Jong, et al. Outbreak of Salmonella Stanley in Sweden associated with alfalfa sprouts, July–August 2007. Wkly Releases 1997–2007. 2007;12. 17997915

[23] Ding H, Fu T, Smith MA. Microbial contamination in sprouts: how effective is seed disinfection treatment? J Food Sci. 2013;78. 10.1111/1750-3841.12064 23464679

[24] Iwu CD, Okoh AI. Preharvest Transmission routes of fresh produce associated bacterial pathogens with outbreak potentials: a review. Int J Environ Res Public Health. 2019;16:4407. 31717976 10.3390/ijerph16224407 PMC6888529

[25] Fahey JW, Ourisson PJ, Degnan FH. Pathogen detection, testing, and control in fresh broccoli sprouts. Nutr J. 2006;5:13. 16630354 10.1186/1475-2891-5-13 PMC1523358

[26] Regulation (EC) No 852/2004 on the hygiene of foodstuffs. Official Journal of the European Union. European Parliament and Council; 2004.

[27] Regulation (EC) No 2073/2005 on microbiological criteria for foodstuffs. Official Journal of the European Union. European Commission; 2005.

[28] Proposed protocol for whole genome sequencing-based analysis for detection and tracing of epidemic clones of antimicrobial resistant Salmonella and Campylobacter—to be used for national surveillance and integrated outbreak investigations by NRLs for public health. FWD AMR-RefLabCap; 2022.

