# Supplementary material for: A large outbreak of multiple Salmonella serovars linked to alfalfa sprouts in Norway, October to December 2024
Source: Infection. 2025 May 21;53(5):2125–34. doi: 10.1007/s15010-025-02556-2 (PMC12460573; doi:10.1007/s15010-025-02556-2)

**Supplementary material for the article “A large outbreak of multiple Salmonella serovars linked to alfalfa sprouts in Norway, October to December 2024”**

**Supplementary Appendix 1** Description of ISO 6579-1:2017 Protocol for Microbiological Testing

| Microbiological analyses were conducted by NRL-*Salmonella* at NVI using VIDAS ®UP Salmonella (bioMerieux) and ISO 6579-1:2017/Amd.2020. For the ISO method, the samples were enriched in buffered peptone water (BPW-ISO Oxoid CM1049) at 37°C for 18 ± 2hrs. After incubation, 1.0 ml and 0.1 ml of BPW were transferred to MKTTn (Muller-Kauffman-Tetrathionate-novobiocin broth, BDH Chemicals 84624) and RVS (Rappaport Vasiliadis Soy broth, Oxoid CM866), respectively, for selective enrichment. The MKTTn and RVS tubes were incubated at 37°C and 41.5 ± 1°C for 24 ± 3 hrs respectively. Aliqouts of 10 µl from each selective enrichment were plated on XLD (Xylose Lysine Deoxycholate agar, Oxoid CM 469) and BGA (Brilliant Green Agar, Merck Millipore 1.10747) and incubated at 37°C for 24 ± 3 hrs. To improve sensitivity, a third selective enrichment procedure was used by adding a tube of MKTTn broth incubated at 41.5 ± 1°C. Typical *Salmonella* colonies were confirmed using MALDI-TOF MS (Bruker), and serotyped by seroagglutination according to ISO 6579-3:2014. |
| --- |

**Supplementary Table S1** Descriptive characteristics of cases in the national multi-serovar *Salmonella* outbreak in Norway, October–December 2024.

| **Descriptive characteristics** | **Cases (n=230)** |
| --- | --- |
| Age (years)^1^ | 48 (0 -90) |
| Age group |  |
| 0-19 | 14 (6.1%) |
| 20-39 | 68 (30%) |
| 40-59 | 79 (34%) |
| 60-79 | 60 (26%) |
| > 80 | 9 (3.9%) |
| Sex |  |
| Men | 71 (30.9%) |
| Women | 159 (69.1%) |
| Hospitalisation | 76 (33.0%) |
| County of residence |  |
| Agder | 7 (3.0%) |
| Akershus | 24 (10%) |
| Buskerud | 7 (3.0%) |
| Finnmark | 5 (2.2%) |
| Innlandet | 15 (6.5%) |
| Møre og Romsdal | 13 (5.7%) |
| Nordland | 2 (0.9%) |
| Oslo | 18 (7.8%) |
| Østfold | 11 (4.8%) |
| Rogaland | 12 (5.2%) |
| Telemark | 11 (4.8%) |
| Troms | 3 (1.3%) |
| Trøndelag | 19 (8.3%) |
| Vestfold | 11 (4.8%) |
| Vestland | 72 (31%) |
| Serovars |  |
| *S*. Newport ST31 cluster 1 (CT25548) | 26 (11.3%) |
| *S.* Newport ST31 cluster 2 (CT26172) | 79 (34.3%) |
| *S.* Typhimurium ST36 | 120 (52.2%) |
| *S*. Kisarawe ST5805 | 3 (1.3%) |
| *S*. Kinondoni ST5447 | 2 (0.9%) |

^1^ Median (min-max)

**Supplementary Table S2** Summary of international investigations of the national multi-serovar *Salmonella* outbreak: EpiPulse notifications and RASFF alerts tracing sprout seeds to the same Italian supplier^a^

| **Countries** | **EpiPulse** | **RASFF** | **Human isolate** | **Food isolate** |
| --- | --- | --- | --- | --- |
| Norway | 2024-FWD-00116 | 2024.9155 | *S*. Newport (ST31)  *S*. Typhimurium  *S*. Kisarawe  *S*. Kinondoni | *S*. Kisarawe  *S*. Kotte  *S*. Newport (ST31)  *S*. Newport (ST166)  *S*. Kinondoni |
|  | 2024-FWD-00049 | - | *S*. Typhimurium | - |
|  | 2024-FWD-00089 | - | *S*. Hvittingfoss | - |
| Sweden | 2024-FWD-00108 | 2024.8840 | *S*. Typhimurium  *S*. Richmond | - |
| Finland | 2023-FWD-0069 | 2023.6714 | *S*. Enteritidis | *S*. Enteritidis |
|  | - | 2025.0200 | - | *S*. Kisarawe |
| Germany | 2023-FWD-0071 | - | *S*. Enteritidis | - |
|  | 2023-FWD-0017 | 2023.4749 | *S*. Adelaide | *S*. Adelaide |
| Italy | - | 2025.0402 | - | *S*. Richmond  *S*. Kinondoni |
| Spain | - | 2024.7982 | No outbreak investigation | *Salmonella* sp. |
|  | - | 2024.8206 |  |  |

^a^ *Information based on ECDC-EFSA Rapid Outbreak Assessment, 2025, on the multi-serovar* *Salmonella* *outbreak linked to sprouted seeds.*

**Supplementary Fig. S1** Age and sex distribution of cases in the national multi-serovar Salmonella outbreak in Norway, October–December 2024


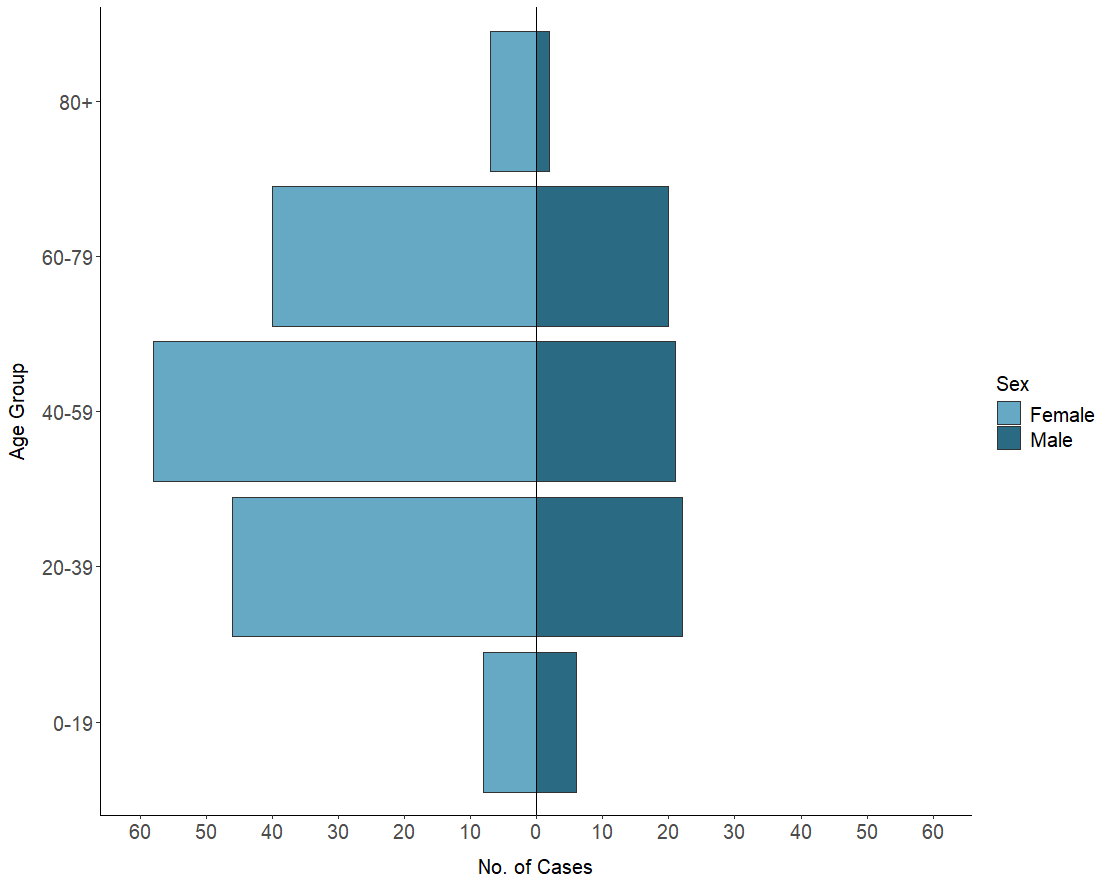


**Supplementary Fig. S2** Geographical distribution of the cases in the national multi-serovar *Salmonella* outbreak by county of residence, Norway, October–December 2024


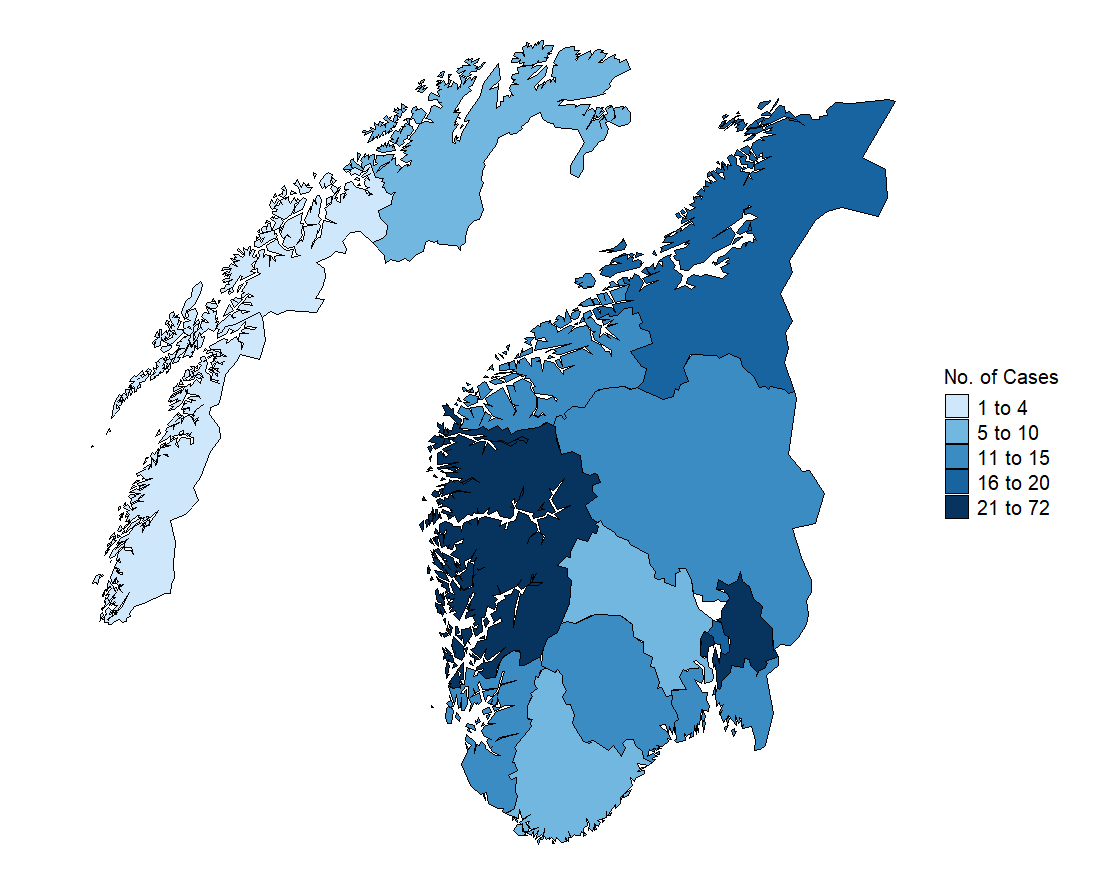

Supplement: Supplementary file 1 — Supplementary file1 (DOCX 89 KB) [file 15010_2025_2556_MOESM1_ESM.docx]
